# Blood Transfusion Knowledge among Nurses in Malaysia: A University Hospital Experience

**DOI:** 10.3390/ijerph182111194

**Published:** 2021-10-25

**Authors:** Noor Haslina Mohd Noor, Noor Hafiza Saad, Mohammad Khan, Mohd Nazri Hassan, Marini Ramli, Rosnah Bahar, Shafini Mohamed Yusoff, Salfarina Iberahim, Wan Suriana Wan Ab Rahman, Zefarina Zulkafli, Md Asiful Islam

**Affiliations:** 1 Department of Haematology, School of Medical Sciences, Universiti Sains Malaysia, Kubang Kerian 16150, Kelantan, Malaysia; nazrihas@usm.my (M.N.H.); marini@usm.my (M.R.); rosnahkb@usm.my (R.B.); shafini@usm.my (S.M.Y.); salfarina@usm.my (S.I.); zefarina@usm.my (Z.Z.); 2 Transfusion Medicine Unit, Hospital Universiti Sains Malaysia, Kubang Kerian 16150, Kelantan, Malaysia; 3 Department of Pathology, Hospital Umum Sarawak, Kuching 93586, Sarawak, Malaysia; norhafizasaad@gmail.com; 4 Universiti Sains Malaysia, Kubang Kerian 16150, Kelantan, Malaysia; drmohammadkhan1001@gmail.com; 5 School of Dental Sciences, Universiti Sains Malaysia, Kubang Kerian 16150, Kelantan, Malaysia; suriana@usm.my

**Keywords:** blood, transfusion, knowledge, nurses, assessment, questionnaire

## Abstract

Blood transfusion is a fundamental and life-saving procedure where the consequence of errors can be fatal. Nurses’ knowledge plays an essential role in ensuring quality and safety in blood transfusion. The objective of this study was to assess blood transfusion-associated knowledge of tertiary hospital nurses on the east coast of Malaysia. This was a cross-sectional study with 200 registered nurses involved in blood transfusion procedures at Hospital Universiti Sains Malaysia. The knowledge of the nurses was evaluated by using the routine blood transfusion knowledge questionnaire based on five parts, and <50%, 50–74%, or ≥75% of the knowledge was considered as poor, moderate, or high, respectively. Based on the scoring system, the overall knowledge of blood transfusion among Malaysian nurses (33.2 ± 8.4 years) was estimated to be 54.9 ± 7.6%. In individual items, the scoring was 81.0%, 45.4%, 49.2%, 63.0%, and 90.0% in knowledge prior to blood transfusion, on pre-transfusion, on post-transfusion, on complications, and on transfusion policy, respectively. The findings of this study indicated that most of the nurses’ overall knowledge of blood transfusion was at a moderate level; therefore, training courses and continuous medical education are warranted to improve knowledge and skills of the nurses to ensure good practices of blood transfusion.

## 1. Introduction

Blood transfusion is a vital, life-saving process in patients with both acute and chronic conditions, aiming to replace lost components of the blood. Millions of people all over the world undergo this process every year, and, generally, it is considered as safe, however, not beyond adverse effects including immunological complications, immunomodulation, or transfusion-transmitted infection [1]. Many human error-associated risks have been reported in blood transfusion processes, which comprise approximately 85% of the total preventable hazards [2]. Acute hemolytic reaction is one of these consequences, resulting in fatality that is mainly caused due to ABO incompatibilities [1].

Considering the risks associated with blood transfusion, there is a growing body of research on creating the optimal safety and care to the patients, where, apprising and improving nurses’ knowledge play a vital role. Blood transfusion is a nursing procedure and adequate knowledge plays a critical role in safe and sound practice. Poor knowledge associated with the compatibility test, blood administration delay, and identification of abnormal reactions followed by blood transfusion are considered as some of the key elements in blood transfusion-related errors [3,4]. In Malaysia, there are a few degrees associated with nursing, such as Diploma (3 years), Bachelor’s degree (4 years), and Master’s degree (2–4 years) in nursing with blood transfusion being part of the curricula. Exploring, evaluating, training, and updating the knowledge of nurses have been the new trends in nursing research towards developing an evidence-based clinical practice as nurses need knowledge in order to make appropriate decisions, and sound knowledge is essential to ensure blood transfusion safety along with high-quality patient care [5,6].

To date, there is no study published assessing the knowledge of nurses in blood transfusion in Malaysia. Therefore, the objective of this study was to evaluate the knowledge of Malaysian nurses on blood transfusion.

## 2. Materials and Methods

### 2.1. Study Selection

This was a cross-sectional study where the registered nurses involved in blood transfusion procedures and having a minimum of 6 months of experience were included from Hospital Universiti Sains Malaysia. A total of 200 nurses were involved this study, based on Hijji et al. [4], where SD = 7.20, Precision (∆) = 1.000, and α = 0.050.

### 2.2. The Routine Blood Transfusion Knowledge Questionnaire (RBTKQ)

For this study, we used the modified version of the *Routine Blood Transfusion Knowledge Questionnaire* (RBTKQ) [4], which has a good reliability score of 68.3 based on the Flesch Reading Ease Index [7]. This questionnaire has seven sections including a total of 43 items. Eight items comprising section A are about the demographic and training information of the nurses. Sections B to F have 33 items about the knowledge assessment of blood bag collection from the blood bank and patient preparation prior to blood transfusion, pre- and post-transfusion nursing responsibilities, and complications related to blood transfusion. The last section (section G) consists of two items addressing the knowledge appraisal on the policies and procedures of blood transfusion in the hospital. Each correct response was awarded with 1 point. The item on the identification of the patient was awarded with 5 points due to its multi-step procedure and was of core competency. No point was awarded if two conflicting responses were selected. The maximum possible score for the RBTKQ is 56 points. Finally, the total score was converted into a percentage and, if the scoring was <50%, 50–74%, or ≥75%, the knowledge was considered as poor, moderate, or high, respectively [8].

### 2.3. Data Analyses

All descriptive data were reported as mean and percentages with a 95% confidence interval (CI). Continuous data were described as mean ± standard deviation (SD). A *p*-value of <0.05 was defined as the level of statistical significance. Data analyses were performed using the Statistical Package for Social Sciences (SPSS) version 27 software (IBM Corporation, Armonk, NY, USA). A simple linear regression was performed to explore the associated factors between gender, qualification, training program, type of ward, working duration, frequency of performing blood transfusion, and mean knowledge score.

### 2.4. Ethics

Ethics were approved from the Human Research Ethics Committee, Universiti Sains Malaysia (USM/JEPeM/140120487). All the nurses received the participant information sheet and signed the informed consent form.

## 3. Results

### 3.1. Characteristics

Detailed characteristics of the nurses are presented in Table 1. In brief, among the participant nurses (33.2 ± 8.4 years), a majority of them were of Malay race (98.0%) and female (94.5%). Most of the nurses possessed a diploma (92.0%) with more than 5 years of working experience (68.5%); however, there was a lack of training experience related to blood transfusions in 92.5% of the participants.

### 3.2. Overall Knowledge

Based on the scoring system, the overall knowledge of blood transfusion among Malaysian nurses was estimated to be 54.9% ± 7.6%, which was considered as a moderate level of knowledge.

### 3.3. Knowledge Prior to Blood Transfusion

**Figure 1:**
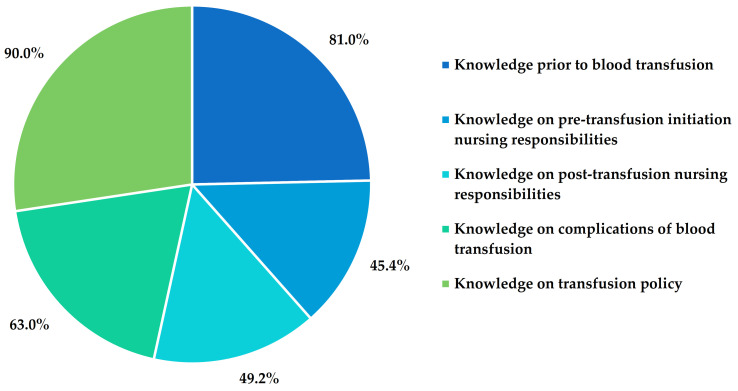
Nurses’ knowledge in different sections of blood transfusion process.

Nurses possessed a high knowledge (>75%) on 80% of the patient preparation and blood pack collection items, except for moderate knowledge on blood bag collection after administration of prescribed pre-medications (68.0%) and ABO basic terminology (67.5%) (Table 2). Overall, in this category, the nurses possessed high level of knowledge (81.2%) (Figure 1).

### 3.4. Knowledge on Pre-Transfusion Initiation Nursing Responsibilities

Interestingly, in the case of knowledge-assessing items under pre-transfusion nursing responsivities, only 18.2% possessed high knowledge, 27.3% had moderate, and 54.5% had poor knowledge (Table 2). A majority of nurses were not aware of clinical indications of blood warming. Overall, about one-third answered correctly for each indication of blood warming (exchange transfusion of infants, rapid transfusion, and patients with cold agglutinin). Most of the nurses (60.5%) knew the best practice of starting the transfusion immediately after bringing a unit of blood to the ward. However, most of the nurses were not sure of blood handling after delivery to the ward from the blood bank. Most of the nurses reported that they would wrap the blood bag with a blanket, allow it to wait at room temperature, immerse it in hot water, and place it in a microwave. A majority of the nurses (64.0%) selected the incorrect filter size. Surprisingly, 26.0% would omit the final bedside identity check in the cases of an unconscious patient, barrier-nursed patient, or when a nurse clearly knows the patient. Overall, in this category, the nurses possessed a poor level of knowledge (45.4%) (Figure 1).

### 3.5. Knowledge on Post-Transfusion Initiation Nursing Responsibilities

Among the questionnaire items related to post-transfusion activities, 44.5% of the nurses had poor knowledge, whereas 33.3% and 22.2% of them possessed moderate and high knowledge, respectively. Among the nurses who worked with adult patients, only half of them were aware of the correct rate to initiate a blood transfusion in adult patients. About three-quarters of the nurses working with pediatric patients were aware of the correct rate to initiate a blood transfusion. A majority of the nurses (91.5%) knew that normal saline is compatible with a blood transfusion (Table 2). Overall, in this category, the nurses possessed a poor level of knowledge (49.2%) (Figure 1).

### 3.6. Knowledge on Complications of Blood Transfusion

Although nurses had a moderate level of knowledge (55.0%) on interventions to minimize the risk of transfusion reactions and signs and symptoms of acute hemolytic transfusion reaction (AHTR) (64.4%), they possessed a high knowledge level (80.1%) on how to manage AHTR (Table 2). Overall, in this category, the nurses possessed a moderate level of knowledge (63.0%) (Figure 1).

### 3.7. Knowledge on Blood Transfusion Policy

Interestingly, in this category, nurses scored a 90.0% overall level of knowledge (Table 2, Figure 1) and a majority of the nurses were aware of the availability of the written policy of blood transfusion in their ward and most of them had read the policy.

### 3.8. Association between Knowledge Score and Characteristics of the Nurses

There was no significant difference of knowledge level in different genders (*p =* 0.466), educational level (*p* = 0.185), types of wards (*p* = 0.897), working experience (*p* = 0.350), and previous training exposure (*p* = 0.177). Interestingly, higher blood transfusion experience in the last 6 months was significantly associated with better knowledge (0 times vs. 1–4 times, *p* = 0.020 and 1–4 times vs. >5 times, *p* = 0.035). From both simple and multiple linear regression models, we observed that nurses who performed blood transfusions between 1 to 4 times (within the past 6 months) had a 4.13% significantly higher (*p* = 0.005) blood transfusion knowledge score (Table 3).

## 4. Discussion

Blood transfusion is one of the high-risk invasive procedures for life-saving practice in various medical conditions. Nurses have a multifunctional role in the blood transfusion process, which requires evidence-based knowledge, various skills, and qualities [9]. Even though having adequate knowledge does not always represent good practice, it is necessary to have a proper education in blood transfusion to ensure patients’ safety and minimize blood transfusion-related hazards [10]. In this study, we assessed the knowledge of a tertiary hospital’s nurses on the east coast of Malaysia on blood transfusion using the *Routine Blood Transfusion Knowledge Questionnaire* (RBTKQ). In our study, most of the respondents were female (94.5%) with Malay ethnicity (98%). Malaysia has a higher rate of female nurses (98.2%) in the hospital, while Malay ethnicity is predominant in our study area of Kota Bharu [11].

This study showed that nurses had a moderate level of overall blood transfusion knowledge (54.9% ± 7.6%). Similarly, previous findings showed that the overall knowledge of nurses about blood transfusion has found to be low to moderate in general [12,13,14,15]. Nearly half of the nurses do not have adequate knowledge of blood transfusion. Knowledge deficits among nurses have been identified in several key aspects of blood transfusion [15].

This study found that nurses have a high knowledge (>75%) on 80% of the patient preparation and blood pack collection items, which is almost similar to a study in Iran (98.4%) [16] and compared to a study in the UAE that reported only 6% of the nurses did establish patency of the venous route before blood collection [17]. More than two-thirds of our nurses (68.0%) understood that the collection of blood bags could be done after any prescribed medication was given with knowledge of ABO basic terminology (67.5%), which compared better than the study conducted by Hijji et al. [15], where only one-third of the nurses were aware of this requirement. Most of our nurses had high knowledge that they should provide patients with the indications and risks of blood transfusion and reaction symptoms. A survey on blood transfusion informed consent among healthcare givers and patients found that transfusion hazards were more likely to be understated [18]. Most nurses at Oxford University Hospital reported that they clarified the need for blood transfusion to the patients [19].

Our study demonstrated that the overall knowledge score on pre-transfusion initiation nursing responsibilities is of a poor level (45.4%). Proper bedside identification of patients who are scheduled for transfusion is important to avoid new errors and identify those errors that may have happened earlier [20]. However, considering that, only two-thirds of our nurses (65.5%) acknowledged that the most important nursing action before transfusion begins was patient identification. Nurses showed better responses about this important step compared to Jordanian nurses, where only 30% had this knowledge [15]. The primary cause of incorrect transfusions that could result in substantial morbidity and mortality of patients is insufficient patient identification. Nurses are also crucially responsible for early patient identification as a key competency [20].

The last chance to detect any anomalies and to prevent any transfusion mistakes is the final bedside checking procedure. Failure to properly perform the final bedside check may lead to the administration of incorrect blood [21]. Our study found that, in cases of an unconscious patient, a barrier-nursed patient, or when nurses know the patient, 26% of nurses will omit the final bedside identity search. This finding is similar to other reported studies where nurses failed to comply with this simple task [4,15,22,23]. These results indicated that, in the most significant steps in the transfusion chain process, there was a lack of understanding and proper knowledge. Some of the factors that promote the incidence of transfusion error were (1) blood monitoring away from the bedside, (2) nursing staff confusion, and (3) transfusion performance in clinical emergencies. The blood-warming procedure is one of the most important steps in transfusion because the increased temperature is associated with hemolysis, which leads to fever, coagulopathy, and renal insufficiency, sometimes causing death [24]. Only one-third of our nurses answered correctly for each indication of blood warming (exchange transfusion of infants, rapid transfusion, and patients with cold agglutinin). To date, many devices such as counter-current heat exchange, dry heat, and thermostatically controlled water baths are equipped with a warning system to avoid excessive heat and are available on the market [25].

Nurses had a poor level of overall knowledge score (49.2%) on post-transfusion-related nursing responsibilities. One-third of our nurses (32%) correctly responded that the flow rate should be set immediately after the start of the blood transfusion. This result is comparable to the research carried out in the UAE and Jordan [4,15]. Most of our nurses (89%) recognized that heart disease patients are indicated for slow blood transfusion, to avoid transfusion-associated circulatory overload [26,27]. Another finding is that only half of the respondents were aware of the correct rate to initiate blood transfusion. Each nurse should understand that, during the first 15 min of setting up a transfusion, the most serious reactions occur [4]. However, nurses may initiate a transfusion at a rate other than a recommended one without proper knowledge. This could result in either prolonging the length of the transfusion with an increased risk of a bacterial infection or in the incidence of a serious transfusion reaction [28].

Blood transfusions are one of hospitals’ most common procedures that have major risks. Thus, the complications associated with blood transfusion must be known by all concerned nurses. Most of our nurses had a moderate level of knowledge about interventions to minimize the risk of transfusion reactions and AHTR. They had a high level of knowledge (80.1%) about how to manage AHTR, similar to the study reported in India and Iran [29,30].

Most of our responding nurses (91%) were aware of the availability of their wards’ written blood transfusion policy, and most of them had read the policy. While half of the nurses had an overall deficit of blood transfusion knowledge, the policy and guidelines already provided the information about the transfusion procedure chain, blood component transfusion indications, transfusion complications, and the role of each staff involved in the transfusion [31]. Another study also showed that nurses have high knowledge regarding blood transfusion policy while there was a major deficit of overall knowledge [15]. In this study, only the frequency of blood transfusion activity among nurses was found to have a major impact on the knowledge score for blood transfusion. For those who also assisted in blood transfusion, as a result of repetitive functional skills, the performance was higher, requiring the nurses to remember their experience and knowledge.

### Limitations and Recommendations

This was the first survey of nurses’ knowledge of blood transfusion in Malaysia with an excellent response rate. Although the sample is small but randomly selected, the results could be used to educate nurses in other Malaysian and overseas hospitals. One of the weaknesses was that nurses may have recorded details that they did not necessarily use in the practice. Knowledge status does not reflect the actual practice of the procedure of blood transfusion. Furthermore, the results established various levels of knowledge deficiencies in all aspects of blood transfusion. For all Malaysian nurses involved in the blood transfusion process, a compulsory ongoing educational program is required for a better outcome. There are various limitations to measuring knowledge. Certain aspects of blood transfusion that future research might include may likely have been overlooked by this questionnaire, such as the signs and symptoms of AHTR (i.e., back or kidney pain). Further studies are warranted to determine the educational effect on the knowledge and practice of blood transfusion by nurses and what they should follow. These are the critical and corrective measures to be taken to protect patients, eliminate preventable risks of blood transfusion, and increase the efficacy of the transfusion process.

## 5. Conclusions

The findings of this study showed that most of the nurses’ overall knowledge on blood transfusion was at a moderate level, comprising a lack of understanding about ABO blood grouping, blood warming, transfusion rate, and possible complications. Immediate actions from the governing authority including the provision of training courses to deliver the appropriate knowledge is warranted. Continuous medical education for the improvement of nurses’ knowledge and skills as well as routine assessments should be carried out to ensure good practice in blood transfusion.

## Figures and Tables

**Table 1:** Characteristics of the participants (n = 200).

Characteristics	n (%)/Mean ± SD
**Age (years)**	33.2 ± 8.4
**Ethnicity**	
Malay	196 (98.0)
Chinese	2 (1.0)
Others	2 (1.0)
**Gender**	
Male	11 (5.5)
Female	189 (94.5)
**Educational level**	
Diploma in Nursing	184 (92.0)
Bachelor’s and master’s in Nursing	16 (8.0)
**Types of wards**	
Medical based	106 (53.0)
Surgical based	94 (47.0)
**Working experience**	
<5 years	63 (31.5)
5–10 years	58 (29.0)
>10 years	79 (39.5)
**Frequency of performing blood transfusion in past 6 months**	
0 times	37 (18.5)
1–4 times	89 (44.5)
>5 times	74 (37.0)
**Previous training related to blood transfusion**	
No	185 (92.5)
Yes	15 (7.5)

SD: standard deviation.

**Table 2:** Nurses’ knowledge on blood transfusion based on the Routine Blood Transfusion Knowledge Questionnaire (n = 200).

Items	Correct	
	n	%
***A. Knowledge prior to blood transfusion***		
Checking patency of IV line before blood bag collection	177	88.5
Blood bag collection after administration of prescribed pre-medications	136	68.0
Decisions to be taken by nurses with incomplete order of blood transfusion	181	90.5
Three aspects of information given to the patients Reasons for blood transfusion Risks of blood transfusion Reaction symptoms	181 152 165	90.5 76.0 82.5
Baseline vital signs’ recording (Within ½ hour before transfusion)	164	82.0
Information to ensure collection of right blood (Patient’s identification details are identical on the blood bag and blood request form).	165	82.5
Blood bag transport method (validated special box)	167	83.5
Knowledge of ABO basic terminology	135	67.5
***B. Knowledge on pre-transfusion initiation nursing responsibilities***		
Most important nursing action before starting transfusion (patient identification)	131	65.5
Clinical indications of blood warming Exchange transfusion in infants Rapid transfusion Patients with cold agglutinins	78 79 61	39.0 39.5 30.5
Best time to start transfusion	121	60.5
Blood handling after delivery to ward (start transfusion immediately)	49	24.5
Steps for patient identification Ask patient to state his/her name Ensure patient identification match on blood bag and request form Ask patient his/her date of birth	156 98 6	78.0 49.0 3.0
Suitable filter size of transfusion set (170–200 micron)	72	36.0
Omitting final bedside identity check (never acceptable)	148	74.0
***C. Knowledge on post-transfusion nursing responsibilities***		
Activities to perform after starting blood transfusion Setting up flow rate Documentation of relevant information Observation of transfusion reactions	64 164 162	32.0 82.0 81.0
Rate to initiate a transfusion in adult patient ^1^ (n = 161)	82	50.9
Regulation of slow transfusion rate ^2^ Electronic Manual	59 41	29.5 70.5
Maximum duration of using blood administration set in continuous multiple transfusion	6	3.0
Rate to initiate blood transfusion in infant ^3^ (n = 39)	29	74.4
Maximum duration to complete a unit of blood	124	62.0
Indications for slow transfusion Patients with heart disease Patients with severe anemia	178 18	89.0 9.0
Agents compatible with blood Normal Saline Morphine	183 6	91.5 3.0
Vital signs’ recording after starting transfusion (start transfusion at 2:00 p.m.) 2:05 p.m. 2:15 p.m. 3:15 p.m. 4:15 p.m.	142 142 60 50	71.0 71.0 30.0 25.0
Timing and duration of when it is essential to physically observe patient	67	33.5
***D. Knowledge on complications of blood transfusion***		
Nursing interventions to minimize risk of transfusion reactions Administration of compatible blood Starting transfusion within 20 min Total transfusion duration not more than 4 h Avoid incompatible drug/solution	150 70 91 129	75.0 35.0 45.5 64.5
Signs and symptoms of acute hemolytic transfusion reaction (AHTR) Tachycardia Chest pain Hypotension Nausea/vomiting	154 153 128 80	77.0 76.5 64.0 40.0
Nursing management of AHTR Stop blood transfusion Keep vein open with Normal Saline Check patient’s vital signs Notify doctor and begin emergency treatment	198 80 180 183	99.0 40.0 90.0 91.5
Actions to do for delay in starting blood transfusion	52	26.0
Usual presenting complaint of mild transfusion reaction	184	92.0
First action to take in mild allergic reaction	3	1.5
Commonest cause of fatal transfusion reaction	67	33.5
Complication of rapid administration of cold blood	114	57.0
***E. Knowledge of transfusion policy***		
Availability of written policy for blood transfusion in the ward No Yes Do not know	8 182 10	4.0 91.0 5.0
Reading the policy No Yes Not indicated	4 178 18	2.0 89.0 9.0

^1^ This was not applicable to 39 nurses (nurses who were not working in adult ward). ^2^ This is not a knowledge score. ^3^ This was not applicable to 161 nurses (nurses who were not working in pediatric ward).

**Table 3:** Associated factors of knowledge score by Simple Linear Regression model (n = 200).

Variables	b^a^ (95% CI)	*p*-Value
Age (years)	0.07 (−0.05, 0.20)	0.259
Gender		
Male	0	0.466
Female	1.73 (−2.94, 6.41)	
**Qualification**		
Diploma	0	0.185
Bachelor and master	−2.64 (−6.56, 1.28)	
**Type of ward**		
Surgical	0	0.897
Medical	0.14 (−2.00, 2.28)	
**Working experience (years)**		
Less than 5	0	
5 to 10	−1.61 (−4.35, 1.13)	0.248
More than 10	0.20 (−2.35, 2.74)	0.878
**Frequency of performing blood transfusion**		
None	0	
1 to 4	4.13 (1.25, 7.01)	0.005
5 and above	1.06 (−1.91, 4.03)	0.481
**Participation in training program**		
Not participate	0	0.117
Participate	3.21 (−0.81, 7.24)	

b^a^: Crude regression coefficients; CI: Confidence Interval.

## Data Availability

The data presented in this study are available in the article.
